# Xylan alleviates dietary fiber deprivation-induced dysbiosis by selectively promoting Bifidobacterium pseudocatenulatum in pigs

**DOI:** 10.1186/s40168-021-01175-x

**Published:** 2021-11-21

**Authors:** Zhenyu Wang, Yu Bai, Yu Pi, Walter J. J. Gerrits, Sonja de Vries, Lijun Shang, Shiyu Tao, Shiyi Zhang, Dandan Han, Zhengpeng Zhu, Junjun Wang

**Affiliations:** 1 State Key Laboratory of Animal Nutrition, College of Animal Science and Technology, China Agricultural University, No. 2 Yuanmingyuan West Road, Beijing, 100193 China; 2 Animal Nutrition Group, Wageningen University & Research, PO Box 338, 6700 AH Wageningen, The Netherlands; 3 Tequ Group Co., Ltd., Chengdu, 611400 Sichuan China

**Keywords:** Bifidobacterium, Gut health, Microbiota, Xylan

## Abstract

**Background:**

Low dietary fiber intake has been shown to disturb the gut microbiome community, damage the mucus barrier, and promote pathogen susceptibility. However, little is known about the temporal response of the gut microbiome to dietary fiber deprivation and the recovery induced by dietary fiber inclusion in pigs.

**Objective:**

In the present study, temporal responses of ileal and fecal microbiota to dietary fiber deprivation were profiled using an ileum cannulated growing pig model. In addition, the potential of dietary-resistant starch, β-glucan, and xylan to alleviate gut dysbiosis throughout the gastrointestinal tract, as well as its possible mechanisms were investigated.

**Methods:**

Six cannulated growing pigs were fed a fiber deprivation diet for 35 days. Ileal digesta and feces were collected at days 0, 7, 21, and 35 for 16S rRNA sequencing and short-chain fatty acid (SCFA) determination. Another twenty-four healthy growing pigs were assigned to one of four dietary treatments including (1) fiber-free diet, (2) resistant starch diet, (3) β-glucan diet, and (4) xylan diet. These twenty-four pigs were fed a corresponding diet for 35 days and slaughtered. Gut microbiome and SCFA concentration were profiled along the gastrointestinal tract.

**Results:**

Dietary fiber deprivation-induced consistent microbiota extinction, mainly *Bifidobacterium* and *Lactobacillus*, and decreased SCFA concentrations in both ileum and feces. The community structure partially recovered at day 35 compared with baseline while SCFA concentrations remained low. Xylan supplementation alleviated gut dysbiosis by selectively promoting *Bifidobacterium pseudocatenulatum* within the large intestine. SCFA concentration increased significantly after xylan supplementation and exhibited a positive association with *B*. *pseudocatenulatum* abundance. An elevated abundance of xylan degradation-related enzyme genes was also observed in the gut microbiome after xylan supplementation. In vitro growth assay further verified the xylan utilization capacity of *B*. *pseudocatenulatum.*

**Conclusions:**

Dietary fiber deprivation could induce probiotic extinction and loss of the SCFA production while potential pathogen was promoted. Xylan intervention could partially restore dietary fiber deprivation-induced gut dysbiosis through selectively promoting *B*. *pseudocatenulatum* and therefore normalizing the gut environment. These findings collectively provide evidence that dietary fiber-driven microbiota metabolism bridges the interplay between microbiome and gut health.

**Video abstract**

**Supplementary Information:**

The online version contains supplementary material available at 10.1186/s40168-021-01175-x.

## Introduction

Trillions of microbes inhabit the gastrointestinal tract of mammals, playing critical roles in gut development, nutrient digestion, immune maturation, and resistance to pathogens [1]. Emerging data shows that gut dysbiosis is associated with various non-infectious diseases [2–4]. Dietary fiber insufficiency has been shown to be associated with gut dysbiosis, therefore leading to gut inflammation, colon cancer, obesity, and type 2 diabetes [5]. However, the specific mechanism underlying these gut microbiota-associated diseases is still unclear. Rodents have been widely employed in gut microbiome research, but several physiological and metabolic differences between rodents and humans have to be acknowledged [6]. The similarity between humans and pigs in terms of intestinal microbial ecosystem and fiber fermentation capacity places the pig in a superior position over other animal models, especially for dietary fiber intervention research [7].

Dietary fiber is a heterogeneous class of components, mainly composed of carbohydrates. It cannot be digested by endogenous enzymes of human beings and other mammals in the small intestine [8]. In contrast, dietary fiber is largely fermented by microbiota inhabiting the large intestine and converted to SCFA, predominantly acetate, propionate, and butyrate [9]. Feeding a fiber-free diet for weeks was reported to thin the mucus layer, increase susceptibility to enteric infection, and lead to the extinction of specific species in mice [10, 11]. Therefore, dietary fiber deprivation has become an effective tool to study the causality between dietary fiber and gut microbiota-associated diseases. To date, knowledge about the response of human gut microbiota to dietary fiber deprivation is still lacking.

Due to the complicated and variable nutrient composition of fiber-rich ingredients, inconsistent experimental outcomes across experiments and correlation rather than causality were generally observed in various studies [12, 13]. Xylan, β-glucan, and resistant starch are the major fraction of fermentable dietary fiber contained in cereal grains and legumes [14]. Deciphering the effect of dietary fiber on gut microbiome using the above purified dietary fiber component will give us mechanistic insights into how these dietary fiber components influence intestine health.

Mammals harbor distinct microbial communities in the small intestine, cecum, and colon, which is thought to be due to local micro-environmental variation including nutrient and chemical gradient as well as compartmentalized host immune activity [15, 16]. Data shows that spatial heterogeneity of gut microbiota along the gastrointestinal tract exerts specialized function in accordance with host physiology [17, 18]. However, studies concerning gut microbiome response to dietary fiber supplementation rely largely on stool samples which are not sufficient to reflect the dynamics response of gut microbiota along the digestive tract. Exploring the spatial response of gut microbiota to dietary fiber will provide detailed and comprehensive information on how dietary fiber interacts with gut microbiota along the gastrointestinal tract.

In the present study, we aimed to investigate the temporal response of ileal and fecal microbiota and metabolites to dietary fiber deprivation with a pig model. Furthermore, the potential role of xylan, β-glucan, and resistant starch re-supplementation to alleviate dietary fiber deprivation-induced gut dysbiosis was evaluated. Metagenome sequencing and in vitro growth assay were included as well to further elucidate the microbiota-associated mechanism. Collectively, our work revealed the physiological changes induced by fiber deprivation and the specific role of xylan in alleviating it, providing a basis for future nutritional intervention for gut dysbiosis.

## Material and methods

### Animals

Experiments were conducted at the swine research unit of China Agricultural University (Chengde, China). Six growing barrows fitted with ileum T-cannula were used in the first experiment. Briefly, six growing barrows were individually housed in stainless steel metabolism crates (1.80 m × 0.65 m). After 7-day adaptation, pigs were fasted for 12 h prior to fitting a simple T-cannula in the distal ileum, approximately 5 cm anterior to the ileocecal valve. The specific surgery operation has been described previously [19]. During the first 3 days after surgery, pigs were fed a specific diet including glucose and multivitamin and then gradually adapted to a standard maize-soybean meal diet for 18 days. After the 21-day recovery period, pigs were fed a fiber-free diet for 35 days. The nutrient composition was listed in Supplemental Table 1. Fresh ileal digesta and feces were collected at days 0, 7, 21, and 35 immediately after ingestion in the morning. All samples were immediately frozen in liquid nitrogen and stored in − 80°C. Sample information was listed in Supplemental Table 2, 3.

Then, 24 growing barrows with an average initiative bodyweight of 59.7 ± 2.6 kg were used in a second experiment and assigned to one of four dietary treatments including (1) fiber-free diet, (2) resistant starch diet, (3) β-glucan diet, and (4) xylan diet. The resistant starch, β-glucan, and xylan were extracted from wheat, oat, and maize, respectively. All diets were formulated to meet or exceed the nutrient requirement for growing pigs (NRC 2012), which was listed in Supplemental Table 1. The experiment lasted for 42 days. All pigs were fed a standard corn-soybean meal diet to eliminate gut background difference for 7 days. At day 8, pigs were switched to corresponding experimental diets and lasted for 35 days. All pigs were individually housed in stainless-steel metabolism crates (1.80 m × 0.65 m) and had ad libitum access to water throughout the experiment. Each metabolism crate was equipped with a feeder, nipple drinker, and slatted floor. The daily feed allowance was set as 2.2 times of maintenance requirement (433 kcal/kg BW^0.6^) according to NRC (2012) [20]. At the end of the experiment, pigs were euthanized by electrical stunning and exsanguination. The cotton rope was first used to prevent the movement of the digesta between intestinal segments. The fresh digesta within the duodenum, jejunum, ileum, cecum, proximal colon, middle colon, distal colon, and feces were collected and immediately frozen in liquid nitrogen and stored at − 80°C for further analysis. The proximal colon, middle colon, and distal colon were defined by dividing the whole colon into three equal segments. All samples were collected using 2-mL centrifuge tubes.

### DNA extraction

Microbial DNA was extracted from luminal content samples using FastDNA SPIN Kit for Soil (MP Biomedicals, USA) according to the manufacturer’s protocol. Extracted DNA was quantified using NanoDrop 2000 (Thermo Fisher Scientific, USA). DNA quality was evaluated by agarose gel electrophoresis. V3–V4 regions of the bacteria 16S rRNA gene was amplified with primer pairs 338F (5′-ACTCCTACGGGAGGCAGCAG-3′) and 806R (5′-GGACTACHVGGGTWTCTAAT-3′) by an ABI GeneAmp 9700 PCR thermocycler (ABI, CA, USA) [17]. The optimized conditions for PCR amplification were as follows: initial denaturation at 95°C for 3 min, 27 cycles of denaturing at 95°C for 30 s, annealing at 55°C for 30 s, and extension at 72°C for 45 s, followed by a final extension at 72°C for 10 min. The 20-μl PCR reaction includes 5 × FastPfu Buffer of 4 μl, dNTPs (2.5 mM) of 2 μl, Forward Primer (5 μM) of 0.8 μl, Reverse Primer (5 μM) of 0.8 μl, FastPfu Polymerase of 0.4 μl, BSA of 0.2 μl, and Template DNA of 10 ng, and ddH_2_O was supplemented to reach a final volume of 20 μl. PCR products were purified, quantified, pooled, and sequenced on the Illumina MiSeq PE300 platform (Illumina, San Diego, USA) according to the standard protocols by Majorbio Bio-Pharm Technology Co. Ltd. (Shanghai, China).

### 16S sequence processing and analysis

16S raw sequencing reads were demultiplexed according to sample-specific barcode (6–8 nucleotides) and imported into QIIME2 platform (version 2020.2) [21]. Quality control and denoising were performed simultaneously using DADA2 with default parameters to generate ASVs [22]. Only ASVs with a minimum abundance of two reads and detected in more than two samples were retained. The phylogenetic tree was generated using the SEPP algorithm against the silva 132 database with default parameters [23]. To avoid the bias resulting from different sequencing depths, all samples were rarefied to 5052 sequences which still yielded average Good’s coverage of 99.70% [24]. All ASVs were classified against the silva 132 database by naïve Bayes classifier constructed by scikit-learn software [25]. α- and β-diversity were calculated using the vegan package (versions 2.5–6) inside R. PCoA was performed using weighted Bray-Curtis and UniFrac distance metrics. PERMANOVA was used to evaluate factors shaping microbiota by using the adonis function of the “vegan” package (999 permutations). Differential taxa were identified by LEfSe (linear discriminant analysis effect size) and further classified against the NCBI 16S rRNA database using blast software [26].

### DNA extraction, library preparation, and metagenome sequencing

Digesta of the large intestine of both dietary fiber deprivation and xylan supplementation were subjected to shotgun metagenomics sequencing. Briefly, total genomic DNA was extracted using the E.Z.N.A Soil DNA Kit (Omega Bio-tek, Norcross, GA, U.S.). Concentration and purity were determined with TBS-380 and NanoDrop2000, respectively. The quality of extracted DNA was checked on 1% agarose gel. Then, DNA was fragmented to approximately 400 bp using Covaris M220 (Gene Company Limited, China). Adapter ligation, cleanup, and enrichment were performed using NEXTFLEX Rapid DNA-Seq (Bioo Scientific, USA). Shotgun metagenomic sequencing was performed on Illumina NovaSeq/Hiseq Xten at Majorbio Bio-Pharm Technology Co., Ltd. (Shanghai, China). Quality control was performed using fastp (version 0.19.4) to trim adapters and filter low-quality reads with the parameter “--cut_by_quality3 -W 4 -M 20 -n 5 -c -l 50 -w 3” [27]. Bowtie2 (version 2.4.1) was used to remove reads aligned to the swine genome [28].

### Taxonomy profiles

Microbial taxonomic profiles were generated using MetaPhlAn3 (version 3.0.7) [29]. MetaPhlAn3 relies on approximately 1.1 M unique clade-specific marker gene identified from around 100,000 reference genomes (99,500 bacterial and archaeal and 500 eukaryotic), allowing unambiguous taxonomic assignments, accurate estimation of organismal relative abundance and species-level resolution for bacteria, archaea, eukaryotes, and viruses. Differential species between treatments were identified using LEfSe with an LDA threshold of 4.0. A random forest classification algorithm was used to identify taxonomy biomarkers with ntree = 10000. To estimate the minimal number of top discriminatory taxa required for prediction, the rfcv function implemented in the “randomForest” package was performed 5 times.

### Metagenomics assembly and functional annotation

The filtered high-quality reads were co-assembled using MEGAHIT (version 1.2.9) using the option “--min-count 2 --min-contig-len 500.” [30]. Gene prediction from assembled contig was predicted using prodigal (version 2.6.3) with mode “-meta” [31]. A total of 6,709,811 genes were identified. A custom script was used to remove incomplete genes and 2,886,465 complete genes were retained. Briefly, prodigal provides “partial = 00” in gene id description for each complete gene, we extract id of a complete gene using “grep” and retain corresponding complete gene sequence using seqkit software [32]. A non-redundant gene catalog was constructed using CD-HIT (version 4.6.1) at the protein level with 90% sequence identity and 90% coverage [33]. High-quality reads were mapped back to the constructed non-redundant gene catalog with 95% identity using salmon (version 2.21) to calculate gene abundance within each sample [34]. The clustered amino acid sequences in the gene catalog were then aligned to the dbCAN2 database by diamond (version 2.0.6) to annotate carbohydrate-active enzymes (CAZy) profile [35].

### Growth experiments of bacteria

*B. pseudocatenulatum* DSM 20438 was routinely grown in MRS medium to determine its utilization capacity of different carbon sources. MRS medium was supplemented with sterilized 2% (w/v) carbohydrates. Cultures (15 ml) were grown in triplicates and optical density at 600 nm (OD600) was determined to assess bacterial growth until the stationary phase was reached.

### Xylanase activity

Cell-associated and secreted xylanase activity were determined by growing *B. pseudocatenulatum* DSM 20438 in 15 ml MRS containing 2% (w/v) xylose, xylo-oligosaccharides, xylan, or glucose for 16 h. Cells were harvested (5000 g for 5 min), resuspended in PBS to OD600 = 0.3. Xylanase activity was determined using EnzChek Ultra Xylanase Assay Kit (Invitrogen) according to the manufacturer’s protocol. Xylanase from Aspergillus niger was used as a standard xylanase reference. 10 U/mL solution of control xylanase and 50 μg/mL xylanase substrate were used. The specific protocol was as follows: First, dilute the xylanase-containing samples in 1X Reaction Buffer provided by the kit. Five microliters of the sample preparations was added into microplate wells. To establish a standard curve of known xylanase activity, 400 mU/mL working solution of control xylanase was prepared and diluted serially in a two-fold gradient using 1X Reaction Buffer, then add 50 μL of the final preparation into microplate wells. Next, add 50 μL of the 50 μg/mL xylanase substrate working solution into each microplate well simultaneously using a multichannel pipettor. 50 μL of 1X Reaction Buffer was used as a negative control. The samples were incubated at room temperature for 30 min. The fluorescence was measured using excitation at 360 nm and emission detection at 460 nm.

### SCFA concentration determination

SCFA concentration of luminal content was measured following the previous protocol [36]. Briefly, samples were thawed on ice and approximately 0.5 g sample was added to 8 mL of deionized water. Then, the mixture was thoroughly homogenized by vertexing for 1 min and centrifuged at 13,000 × *g* for 5 min. The supernatant was diluted 50 times and filtered through a 0.22-μm filter (Millipore, Bedford, OH). Extracted sample solution (25 μL) was analyzed by a high-performance ion chromatography of ICS-3000 (Dionex, USA).

To determine the SCFA concentration of the bacteria medium, 1 mL medium was collected at each time point and centrifuged at 13,000 × *g* for 5 min. The supernatant was diluted 100 times and filtered through a 0.22-μm filter (Millipore, Bedford, OH). Other procedures were the same as above.

### Statistical analysis

Statistical analysis was performed using R software (version 3.6.3). α-diversity was analyzed using Kruskal-Wallis test. PCoA was analyzed employing PERMANOVA. ANOVA was used for SCFA concentration and mRNA expression data. DEseq2 package was used to identify differential genes between treatments with FDR *P* value adjustment.

## Results

### Temporal responses of ileal and fecal microbiota to dietary fiber deprivation

**Fig. 1:**
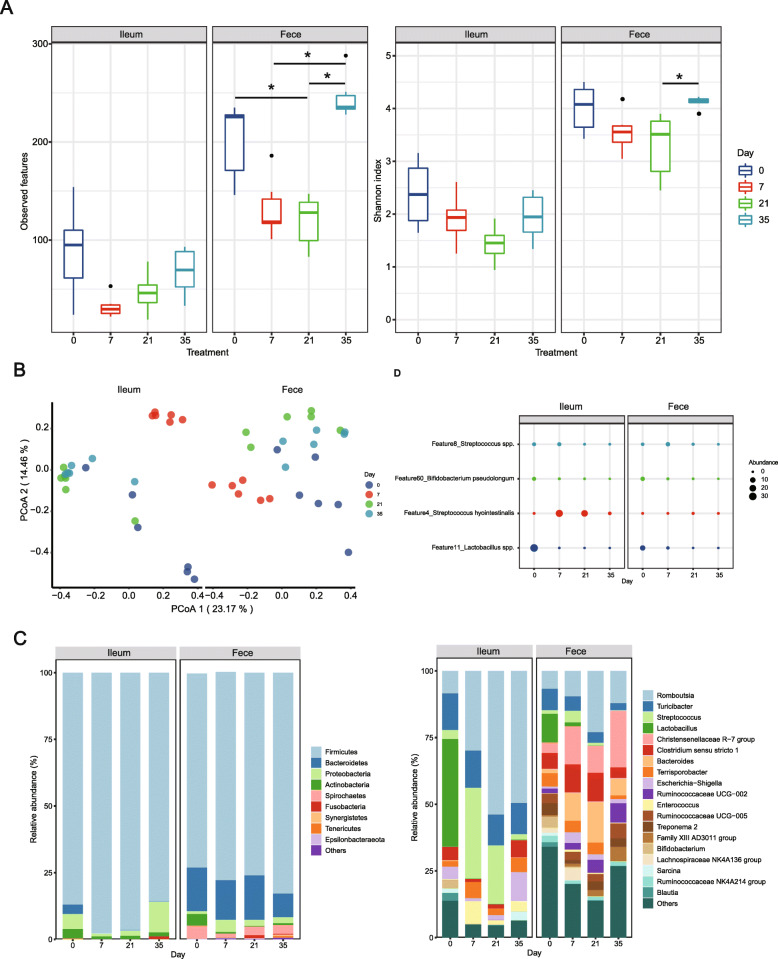
Temporal response of ileal and fecal microbiome to dietary fiber deprivation after adaptation to standard corn-soybean meal diet. **A** Boxplots of alpha diversity as measured by Observed Species and Shannon diversity index of the ileal and fecal microbiome. **B** PCoA of the ileal and fecal microbiome over time based on weighted the unifrac distance metrics. **C** Relative abundance of bacteria at phylum and genus level. The top 10 most abundant phylum and top 20 most abundant genera are shown. **D** Differential abundant ASVs over time. Statistical analyses were performed using Kruskal-Wallis test with *P* value adjustment using FDR correction. Significance between community structure was evaluated by PERMANOVA. ^*^P < 0.05

The overall study design was explained in Supplementary Figure 1. In experiment 1, we profiled the temporal response of ileal and fecal microbiota to dietary fiber deprivation. Overall, α-diversity of feces including Shannon diversity and observed species decreased at days 7 and 21 and significantly increased at day 35, but no significant difference was observed for ileum (Fig. 1A). Clear community shift was observed in feces on day 7 but partially recovered at days 21 and 35 compared to day 0 (PERMANOVA, *P* < 0.05, Fig. 1B). At the phylum level, Firmicutes dominated both ileum and feces accounting for over 75% relative abundance followed by Proteobacteria and Bacteroidetes (Fig. 1C).

In line with the changes of α-diversity, the relative abundance of Proteobacteria decreased at days 7 and 21 and recovered at day 35. Interestingly, dietary fiber deprivation resulted in the extinction of *Lactobacillus* and *Bifidobacterium* genus at day 7, and no recovery was observed thereafter. In contrast, *Enterococcus* and *Streptococcus* were enriched following dietary fiber deprivation. Further analysis by LEfSe revealed that two ASVs classified as *Lactobacillus spp.* (ASV 11) and *Bifidobacterium spp.* (ASV 60) depleted following dietary fiber deprivation. *Streptococcus hyointestinalis* (ASV 4) and *Streptococcus spp.* (ASV 8) enriched at days 7 and 21 and decreased at day 35 (Fig. 1D).

**Fig. 2:**
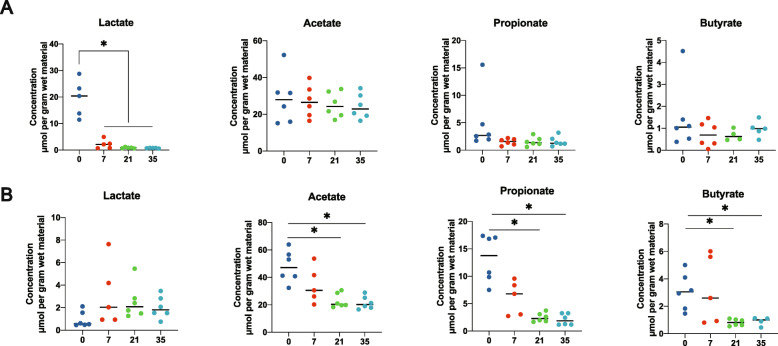
SCFA concentration of the ileum digesta and feces over time. **A** SCFA concentration of the ileum digesta over time following dietary fiber deprivation. **B** SCFA concentration of feces over time following dietary fiber deprivation. Statistical analyses were performed using one-way ANOVA. ^*^P < 0.05

SCFA concentration was also measured at each timepoint. Lactate concentration decreased dramatically at day 7 and then remained constant in the ileum (Fig. 2A). However, different patterns were observed among the other three fatty acids (acetate, propionate, and butyrate). A significant decrease was only observed at days 21 and 35 in feces (Fig. 2B).

### Community diversity and structure along gastrointestinal tract following xylan, β-glucan, and resistant starch re-supplementation

**Fig. 3:**
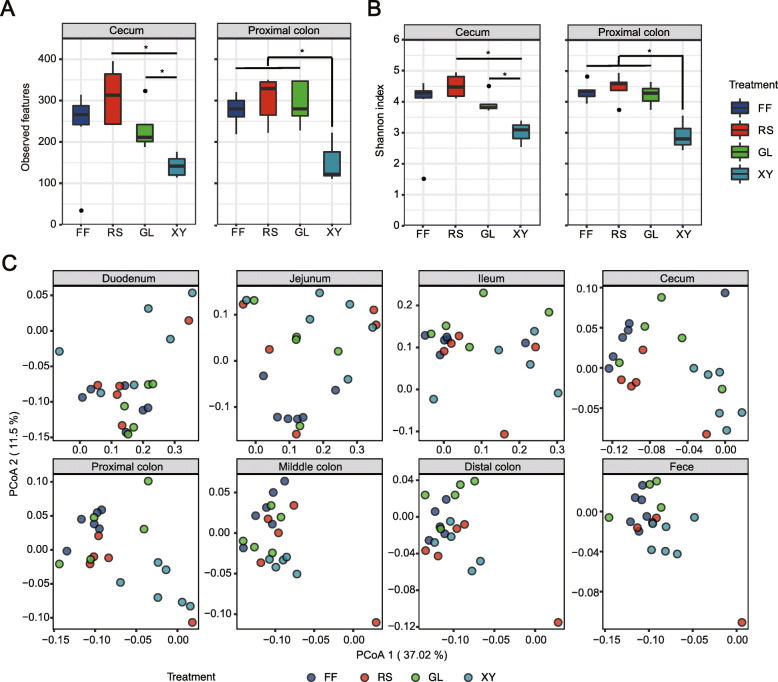
Overall community diversity and structure of gut microbiome following xylan, β-glucan, and resistant starch intervention. **A** Observed species of gut microbiota at the cecum and proximal colon. **B** Shannon index of gut microbiota at cecum and proximal colon. **C** PCoA of gut microbiota along gastrointestinal tract based on weighted unifrac distance metrics. FF dietary fiber deprivation, RS resistant starch, BG β-glucan, XY xylan. Statistical analyses were done using Kruskal-Wallis test with *P* value adjustment using FDR correction. Significance between community structures was evaluated by PERMANOVA. ^*^P < 0.05

In experiment 2, we supplemented a fiber-free diet with 5% fermentable dietary fiber including resistant starch, β-glucan, and xylan. Gut microbiota alteration throughout the gastrointestinal tract was characterized using 16S rRNA amplicon sequencing.

the small intestinal digesta exhibited higher inter-individual variation of α-diversity including observed ASVs and Shannon index compared with that of the large intestine (Supplemental Figure 2A, B). The high variation was reduced while microbial diversity increased from the small intestine to the large intestine. Interestingly, a significant lower α-diversity was observed at the cecum and proximal colon with xylan supplementation (Fig. 3A, B).

We then analyzed the microbiome structure along the gastrointestinal tract using PCoA and PERMANOVA based on weighted unifrac and Bray-Curtis distance metrics. Distinct separation was observed in community structure between the small intestine and large intestine regardless of dietary treatments (PERMANOVA, *P* < 0.05, Supplemental Figure 3A, B). Consistent with the results of α-diversity, xylan supplementation induced significant microbiota shifts within the large intestine, resulting in a clear separation between xylan and other three dietary treatments (PERMANOVA, *P* < 0.05, Fig. 3C; Supplemental Figure 4).

**Fig. 4:**
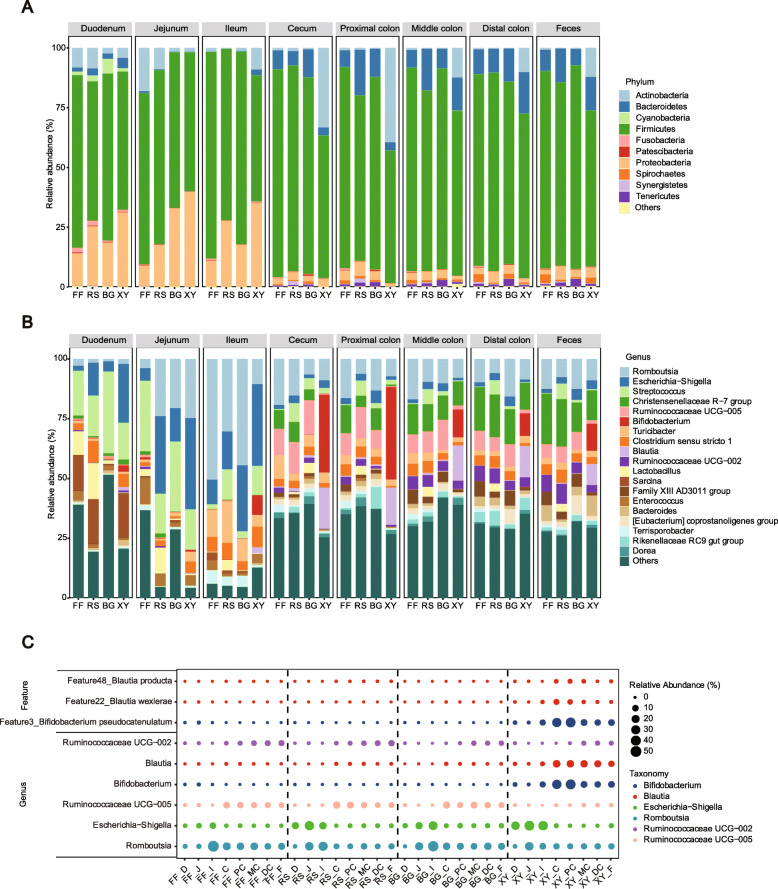
Spatial distribution of microbial community along the gastrointestinal tract following xylan, β-glucan, and resistant starch intervention. **A** The mean relative abundance of top 10 phylum along the gastrointestinal tract. **B** The mean relative abundance of top 20 genera along the gastrointestinal tract. **C** The bubble plot shows the most differential taxa between treatment at genus and ASV level. The size of the bubble is proportional to the relative abundance. FF dietary fiber deprivation, RS resistant starch, BG β-glucan, XY xylan

A total of 24 phyla including Firmicutes, Proteobacteria, Bacteroidetes, and Actinobacteria were detected across all samples (Fig. 4A). Firmicutes and Proteobacteria constituted the two most abundant phyla accounting for over 80% of the total sequences within the small intestine. However, in the large intestine, Proteobacteria were largely replaced by Bacteroidetes and Actinobacteria. At the genus level, the small intestine harbored a high abundance of *Romboutsia*, *Escherichia − Shigella*, and *Streptococcus*, especially in the ileum. In the large intestine, the community was more diverse with no obvious dominant genus (Fig. 4B).

### Xylan selectively alters community composition

Substrate-specific ASVs were identified by LEfSe across all samples at the genus level. The LDA score threshold was set at 4.0. Consistent with the observations above, xylan significantly increased the relative abundance of *Bifidobacterium* and *Blautia* within the large intestine and *Escherichia − Shigella* in the small intestine (Fig. 4C). *Lactobacillus* was most enriched most in the duodenum of fiber-free and resistant starch treatment. The genus *Ruminococcaceae UCG-002* and *Ruminococcaceae UCG-005* showed high abundance across the large intestine except in xylan supplementation treatment. *Romboutsia* was mainly enriched in the small intestine among all treatments.

We further performed LEfSe using 1000 most abundant ASVs which accounted for average of 96.3% sequences of all samples to identify the most differential taxa. By this approach, we identified several ASVs closely associated with xylan supplementation. ASV 3, classified as *Bifidobacterium pseudocatenulatum* with 100% similarity by BLAST, showed extremely high abundance in the cecum and proximal colon (mean abundance 35.31%) when supplemented with xylan (Fig. 4C).

**Fig. 5:**
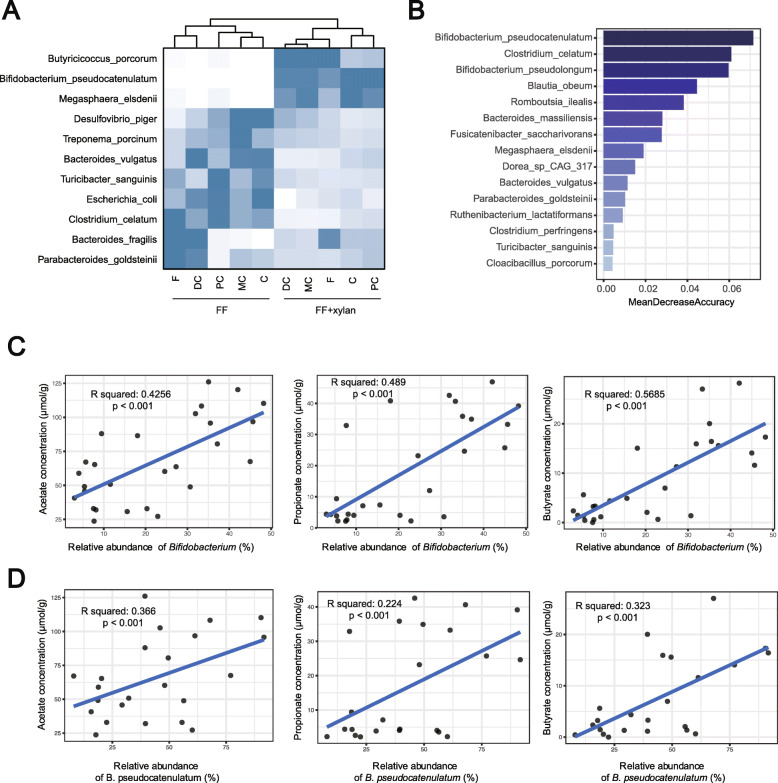
*Bifidobacterium pseudocatenulatum* is a biomarker discriminating dietary fiber deprivation and xylan. **A** The most differential taxa at species level were exhibited by LEfSe analysis using shotgun metagenomic sequencing. **B** Random forest algorithm shows *Bifidobacterium pseudocatenulatum* is the most important taxon discriminating dietary fiber deprivation and xylan. **C** SCFA concentration and its association with a relative abundance of *Bifidobacterium* within the large intestine. **D** SCFA concentration and its association with a relative abundance of *B. pseudocatenulatum* within the large intestine. FF dietary fiber deprivation, FF + xylan, dietary fiber deprivation + xylan. **C** cecum, PC proximal colon, MC middle colon, DC distal colon, F feces

Shotgun metagenome sequencing was employed to further classify taxonomy at the species level. *B. pseudocatenulatum* was shown to be the dominant species promoted by xylan in the proximal colon by LEfSe analysis (Fig. 5A). A random forest classification model was also applied to find bacteria biomarkers discriminating dietary fiber deprivation and xylan supplementation. *B. pseudocatenulatum* was the most important species based on the mean decrease accuracy index (Fig. 5B). This observation was confirmed by using ten-fold cross-validation for five replicates.

### Correlation between *B. pseudocatenulatum* abundance and SCFA concentration

To determine whether xylan-induced shifts in the output of SCFAs were linked to specific bacterial taxa, we conducted regression analysis between the relative abundance of genera *Bifidobacterium* and SCFA concentration (Fig. 5C). Strikingly, a significant linear relationship was detected between *Bifidobacterium* and concentration of acetate, propionate, and butyrate (Fig. 5D). At the species level, similar results were observed between *B. pseudocatenulatum* and the concentration of acetate, propionate, and butyrate.

### Shift in carbohydrate-active enzyme (CAZ) profile of gut microbiome within the large intestine in response to dietary fiber deprivation and xylan

**Fig. 6:**
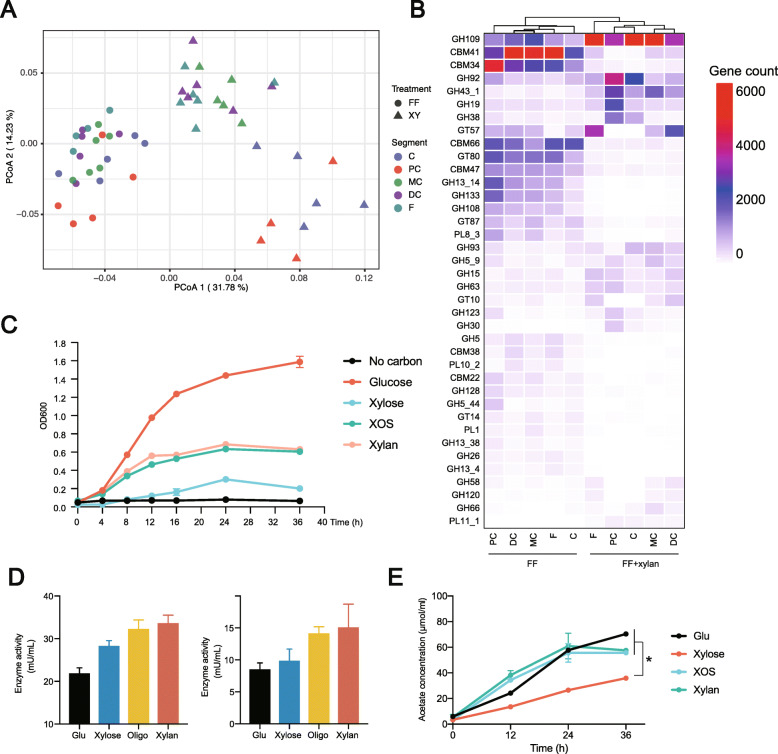
Shift in carbohydrate active enzyme and in vitro assessment of xylan utilization by representative *B. pseudocatenulatum*. **A** An ordinate analysis of CAZ profile. **B** The most differential carbohydrate active enzyme identified by DEseq2. **C** Growth curve (mean OD_600_) of *B. pseudocatenulatum* DSM 20438 in MRS medium containing either 2% of the indicated carbon source or no carbohydrate (control). **D** xylanase activity of *B. pseudocatenulatum* DSM 20438 in MRS medium containing 2% of the indicated carbon source. **E** Acetate concentration of *B. pseudocatenulatum* DSM 20438 in MRS medium containing 2% of the indicated carbon source. FF dietary fiber deprivation, FF + xylan dietary fiber deprivation + xylan. **C** cecum, PC proximal colon, MC middle colon, DC distal colon, F feces, XOS xyl-oligosaccharides. Statistical analyses were done using one-way ANOVA. ^*^P < 0.05

As a relative abundance of *B. pseudocatenulatum* is significantly correlated with SCFA concentration, a shift in carbohydrate-active enzyme (CAZ) profile within the large intestine was characterized. PCoA plot revealed distinct functional profiles between dietary fiber deprivation and xylan treatment groups (PERMANOVA, *P* < 0.05, Fig. 6A). Consequently, we identified differentially represented CAZ families in groups with and without xylan supplementation by using DESeq2. Several starch degrading enzymes (e.g., CBM41, CBM34) were enriched in the dietary fiber deprivation group. In contrast, genes annotated as galactosaminidase, mannosidase, xylanase, and xylosidase (e.g., GH43, GH109, GH92) were mainly found in samples of xylan supplementation treatment, especially in the cecum and proximal colon (Fig. 6B).

### In vitro verification of xylan utilization by *B. pseudocatenulatum*

Due to the failure of isolating *B. pseudocatenulatum* from intestine digesta samples, we used culture collection strain in following experiment. In vitro growth assay shows *B. pseudocatenulatum* DSM 20438 was able to utilize xylose, xylo-oligosaccharides, and xylan. Based on maximum OD_600_, *B. pseudocatenulatum* DSM 20438 had a higher capacity of utilizing xylo-oligosaccharides and xylan compared with xylose (Fig. 6C). Enzyme activity of xylanase of different carbon sources was also determined. Xylanase activity was highest with xylan as the sole carbon source, which was in support of the results from the growth assay (Fig. 6D). *B. pseudocatenulatum* produces high concentration acetate with xylose, xylo-oligosaccharides, and xylan as carbon sources (Fig. 6E).

## Discussion

The health-promoting benefits of dietary fiber have been purported for decades, but the complicated interaction between dietary fiber with variable physicochemical forms and gut microbiota has not been studied in detail yet [9]. Previous work generally employed feed ingredients to study the beneficial effect of dietary fiber in pigs but the knowledge of the specific effect of a single dietary fiber component was lacking [12, 13, 37]. In the present study, we first investigated whether there is a negative effect of dietary fiber deprivation on the pig gut microbiome. We found that dietary fiber deprivation-induced extinction of probiotics, such as *Lactobacillus* and *Bifidobacterium*. Then, purified resistant starch, β-glucan, and xylan were evaluated for their potential to alleviate dysbiosis induced by dietary fiber deprivation. The mechanism of this positive effect of xylan is by selectively promoting *B. pseudocatenulatum* within the large intestine according to the present results. Finally, in vitro growth assay further verified the xylan utilization capacity and SCFA production by *B. pseudocatenulatum* DSM 20438.

Dietary fiber deprivation and a western-style diet have been shown to lead to the extinction of specific bacteria in mice [38, 39]. Specifically, dietary fiber deprivation induces reduction of *Bacteroidetes S24-7 family*, *Bifidobacterium*, and expansion of *Ruminococcaceae*. Importantly, the B*acteroidetes S24-7 family* and *Bifidobacterium* possess a large repertoire of genes involved in sensing and hydrolyzing numerous diet- and host-derived complex carbohydrates [40]. The lack of dietary fiber-degrading bacteria, including *Bacteroidetes S24-7 family* and *Bifidobacterium*, is likely the primary driver leading to decreased SCFA concentration. We further revealed that the extinction induced by dietary fiber deprivation did not completely recover, although the community structure partially resembled baseline at day 35.

Interestingly, xylan supplementation led to lower community diversity and distinct community composition in the large intestine compared to dietary fiber deprivation. The dominant effect of xylan on gut microbiota was directed towards two bacterial genera, *Bifidobacterium*, and *Blautia*. Previous studies showed that *Bifidobacterium* possesses specific ABC transporters and corresponding solute binding protein which displayed an exceptionally broad specificity and preference for xylan and xylo-oligosaccharides (XOS), making it superior over other bacteria taxa in xylan and XOS utilization [41]. Further metagenomics analysis found that *B. pseudocatenulatum* was the dominant species promoted by xylan in the large intestine. Genome-scale metabolic models showed *B. pseudocatenulatum*, *B. longum*, and *B. kashiwanohense* make good use of arabinoxylan or xylan [42], but *B. pseudocatenulatum* possess broader carbohydrate utilization capacity and higher function redundancy over *B. longum* and *B. kashiwanohense*, making it superior in xylan utilization efficiency [42].

Of note, several consistent effects were observed between our study and previous work investigating corn bran arabinoxylan, such as an increased relative abundance of *Bifidobacterium* and *Blautia* and higher SCFA concentrations [43]. Specifically, arabinoxylan promotes multiple bacteria taxa including *Bifidobacterium longum*, *Prevotella copri*, and members of *Bacteroides* while xylan is mainly related to the growth of *B. pseudocatenulatum*. It seems more bacteria taxa are necessary to hydrolyze arabinoxylan, and this difference is likely attributed to their structure. Arabinoxylan generally exhibits a relatively high arabinose-to-xylose ratio and high amounts of galactose, suggesting its heavily branched structure with complex side chains [44, 45]. On the contrary, xlyan showed a much lower arabinose-to-xylose ratio, which indicates a low substitution degree and simple side chains. Genomic analyses showed that genes encoding arabinoxylan-degrading glycosidase (e.g., β-xylosidase and α-arabinofuranosidase) are conserved only among *B. longum* strains [46, 47]. Furthermore, a significant positive association between the relative abundance of *B. pseudocatenulatum* and SCFA concentration (acetate, propionate, and butyrate) indicated that *B. pseudocatenulatum* may be an important SCFA producer. By using in vitro growth assay, we verified the xylan utilization and SCFA production capacity of *B. pseudocatenulatum* with xylan as the sole carbon source.

## Conclusion

In summary, dietary fiber deprivation could induce probiotic extinction and loss of SCFA production. In contrast, potential pathogens including the genus *Streptococcus* and *Enterococcus* was promoted. Xylan supplementation but not resistant starch and β-glucan partially alleviated the detrimental effect coming from dietary fiber deprivation through selectively promoting *B. pseudocatenulatum* in the large intestine. The proliferation of *B. pseudocatenulatum* was accompanied by the increased SCFA concentrations, which was most pronounced in the cecum and proximal colon, indicating the intestine segment-specific manner of xylan. In accordance with this observation, xylan utilization-related enzyme abundance is also enriched in xylan supplementation. Finally, the xylan utilization and SCFA production capacity by *B. pseudocatenulatum* were confirmed by in vitro assay. The findings provided fundamental evidence for the beneficial role of xylan inclusion on gut health through modulating gut microbiome and SCFA concentration, providing a basis for future nutritional intervention in a situation of dietary fiber insufficiency.

## Supplementary Information

**Additional file 1.** Supplemental Table 1 Formulation and nutrient composition of experimental diets.

**Additional file 2.** Supplemental Table 2 Sample information of experiment 1.

**Additional file 3.** Supplemental Table 3 Sample information of experiment 2.

**Additional file 4.** Supplemental File 1 Linux script for analyzing metagenomics data.

**Additional file 5.** Figure S1 Experimental protocol.

**Additional file 6.** Figure S2 α-diversity difference between small intestine and large intestine following xylan, β-glucan and resistant starch intervention. D, duodenum; J, jejunum; I, ileum; C, cecum; PC, proximal colon; MC, middle colon; DC, distal colon; F, feces.

**Additional file 7.** Figure S3 Community structure difference between small intestine and large intestine following dietary fiber deprivation, xylan, β-glucan and resistant starch intervention based on weighted bray-curtis (A) and unifrac (B) distance metrics.

**Additional file 8.** Figure S4 Community structure along the gastrointestinal tract following dietary fiber deprivation, xylan β-glucan and resistant starch intervention based on weighted bray-curtis distance metrics. FF, dietary fiber deprivation; RS, resistant starch; BG, β-glucan, XY, xylan.

**Additional file 9.** Figure S5 SCFA concentration within large intestine in response to different treatments. (A) SCFA concentration (μmol/ml wet digesta) within large intestine. Data are represented as means ± SD. *, P < 0.05. FF, dietary fiber deprivation, RS, resistant starch; BG, β-glucan; XY, xylan.

## Data Availability

All sequencing reads have been deposited at the NCBI sequence read archive under BioProject PRJNA665641.

## Abbreviations

ASV: Amplicon sequence variants
IBD: Inflammatory bowel disorder
LDA: Linear discriminant analysis
LEfSe: Linear discriminant analysis effect size
CAZy: Carbohydrate-active enzymes
NRC: National Research Council
ORF: Open reading frame
PCoA: Principal coordinate analysis
PERMANOVA: Permutational multivariate analysis of variance
SCFA: Short-chain fatty acid
XOS: Xyl-oligosaccharides
