# Supplementary material for: Xylan alleviates dietary fiber deprivation-induced dysbiosis by selectively promoting Bifidobacterium pseudocatenulatum in pigs
Source: Microbiome. 2021 Nov 21;9:227. doi: 10.1186/s40168-021-01175-x (PMC8606072; doi:10.1186/s40168-021-01175-x)
Supplement: Supplementary file 2 — Additional file 1. Supplemental Table 1 Formulation and nutrient composition of experimental diets. [file 40168_2021_1175_MOESM2_ESM.docx]

Table S1. Formulation and nutrient composition of experimental diets

| Items | Control | Resistant starch | β-glucan | Xylan |
| --- | --- | --- | --- | --- |
| Corn starch | 63.20 | 57.12 | 57.45 | 57.61 |
| Soybean isolated protein | 18.00 | 18.00 | 18.00 | 18.00 |
| Soybean oil | 3.00 | 3.00 | 3.00 | 3.00 |
| Sucrose | 11.20 | 11.20 | 11.20 | 11.20 |
| Resistant starch | 0.00 | 6.08 | 0.00 | 0.00 |
| β-glucan | 0.00 | 0.00 | 5.75 | 0.00 |
| Xylan | 0.00 | 0.00 | 0.00 | 5.59 |
| L-Lysine-HCI | 0.15 | 0.15 | 0.15 | 0.15 |
| DL-Methionine | 0.12 | 0.12 | 0.12 | 0.12 |
| L-Tryptophan | 0.00 | 0.00 | 0.00 | 0.00 |
| L-Threonine | 0.13 | 0.13 | 0.13 | 0.13 |
| Limestone | 0.10 | 0.10 | 0.10 | 0.10 |
| Dicalcium Phosphate | 2.40 | 2.40 | 2.40 | 2.40 |
| Cr_2_O_3_ | 0.30 | 0.30 | 0.30 | 0.30 |
| NaCl | 0.40 | 0.40 | 0.40 | 0.40 |
| Potassium Carbonate | 0.40 | 0.40 | 0.40 | 0.40 |
| Magnesium Oxide | 0.10 | 0.10 | 0.10 | 0.10 |
| Premix^a^ | 0.50 | 0.50 | 0.50 | 0.50 |
| Total | 100.00 | 100.00 | 100.00 | 100.00 |
| Nutrients composition (%) |  |  |  |  |
| ME (kcal/kg) | 3637.24 | 3415.32 | 3427.37 | 3433.21 |
| CP | 15.26 | 15.26 | 15.26 | 15.26 |
| SID Lys | 0.97 | 0.97 | 0.97 | 0.97 |
| SID Met | 0.29 | 0.29 | 0.29 | 0.29 |
| SID Trp | 0.18 | 0.18 | 0.18 | 0.18 |
| SID Thr | 0.59 | 0.59 | 0.59 | 0.59 |
| Ca | 0.66 | 0.66 | 0.66 | 0.66 |
| P | 0.59 | 0.59 | 0.59 | 0.59 |

^a^ Premix provided the following per kilogram of feed: vitamin A, 12,000 IU; vitamin D, 2,500 IU; vitamin E, 30 IU; vitamin K, 3 mg; vitamin B12, 12 μg; D-pantothenic acid, 10 mg; nicotinic acid, 40 mg; choline chloride, 400 mg; Mn, 40 mg; Zn, 100 mg; Fe, 90 mg; Cu, 8.8 mg; I, 0.35 mg; Se, 0.3 mg.
