# Supplementary material for: Xylan alleviates dietary fiber deprivation-induced dysbiosis by selectively promoting Bifidobacterium pseudocatenulatum in pigs
Source: Microbiome. 2021 Nov 21;9:227. doi: 10.1186/s40168-021-01175-x (PMC8606072; doi:10.1186/s40168-021-01175-x)
Supplement: Supplementary file 3 — Additional file 2. Supplemental Table 2 Sample information of experiment 1. [file 40168_2021_1175_MOESM3_ESM.docx]

**Table S2: Sample information of experiment 1.**

| SampleID | Intestine | Treatment | Segment | Day |
| --- | --- | --- | --- | --- |
| D35F11 | LI | FF | Feces | 35 |
| D35F12 | LI | FF | Feces | 35 |
| D35F13 | LI | FF | Feces | 35 |
| D35F14 | LI | FF | Feces | 35 |
| D35F15 | LI | FF | Feces | 35 |
| D35F16 | LI | FF | Feces | 35 |
| D35I11 | SI | FF | Ileum | 35 |
| D35I12 | SI | FF | Ileum | 35 |
| D35I13 | SI | FF | Ileum | 35 |
| D35I14 | SI | FF | Ileum | 35 |
| D35I15 | SI | FF | Ileum | 35 |
| D35I16 | SI | FF | Ileum | 35 |
| D0F11 | LI | FF | Feces | 0 |
| D0F12 | LI | FF | Feces | 0 |
| D0F13 | LI | FF | Feces | 0 |
| D0F14 | LI | FF | Feces | 0 |
| D0F15 | LI | FF | Feces | 0 |
| D0F16 | LI | FF | Feces | 0 |
| D0I11 | SI | FF | Ileum | 0 |
| D0I12 | SI | FF | Ileum | 0 |
| D0I13 | SI | FF | Ileum | 0 |
| D0I14 | SI | FF | Ileum | 0 |
| D0I15 | SI | FF | Ileum | 0 |
| D0I16 | SI | FF | Ileum | 0 |
| D21F11 | LI | FF | Feces | 21 |
| D21F12 | LI | FF | Feces | 21 |
| D21F13 | LI | FF | Feces | 21 |
| D21F14 | LI | FF | Feces | 21 |
| D21F15 | LI | FF | Feces | 21 |
| D21F16 | LI | FF | Feces | 21 |
| D21I11 | SI | FF | Ileum | 21 |
| D21I12 | SI | FF | Ileum | 21 |
| D21I13 | SI | FF | Ileum | 21 |
| D21I14 | SI | FF | Ileum | 21 |
| D21I15 | SI | FF | Ileum | 21 |
| D21I16 | SI | FF | Ileum | 21 |
| D7F11 | LI | FF | Feces | 7 |
| D7F12 | LI | FF | Feces | 7 |
| D7F13 | LI | FF | Feces | 7 |
| D7F14 | LI | FF | Feces | 7 |
| D7F15 | LI | FF | Feces | 7 |
| D7F16 | LI | FF | Feces | 7 |
| D7I11 | SI | FF | Ileum | 7 |
| D7I12 | SI | FF | Ileum | 7 |
| D7I13 | SI | FF | Ileum | 7 |
| D7I14 | SI | FF | Ileum | 7 |
| D7I15 | SI | FF | Ileum | 7 |
| D7I16 | SI | FF | Ileum | 7 |
