# Supplementary material for: Xylan alleviates dietary fiber deprivation-induced dysbiosis by selectively promoting Bifidobacterium pseudocatenulatum in pigs
Source: Microbiome. 2021 Nov 21;9:227. doi: 10.1186/s40168-021-01175-x (PMC8606072; doi:10.1186/s40168-021-01175-x)
Supplement: Supplementary file 4 — Additional file 3. Supplemental Table 3 Sample information of experiment 2. [file 40168_2021_1175_MOESM4_ESM.docx]

**Table: Sample information of Experiment 2.**

| SampleID | Small intestine or large intestine | Treatment | Intestine segment | Day | 16S rRNA sequencing | Metagenomics sequencing |
| --- | --- | --- | --- | --- | --- | --- |
| DC41 | LI | XY | Cecum | 35 | YES | YES |
| DC42 | LI | XY | Cecum | 35 | YES | YES |
| DC43 | LI | XY | Cecum | 35 | YES | YES |
| DC44 | LI | XY | Cecum | 35 | YES | YES |
| DC45 | LI | XY | Cecum | 35 | YES | YES |
| DC46 | LI | XY | Cecum | 35 | YES | YES |
| DC21 | LI | RS | Cecum | 35 | YES | NO |
| DC22 | LI | RS | Cecum | 35 | YES | NO |
| DC23 | LI | RS | Cecum | 35 | YES | NO |
| DC24 | LI | RS | Cecum | 35 | YES | NO |
| DC25 | LI | RS | Cecum | 35 | YES | NO |
| DC31 | LI | GL | Cecum | 35 | YES | NO |
| DC32 | LI | GL | Cecum | 35 | YES | NO |
| DC33 | LI | GL | Cecum | 35 | YES | NO |
| DC34 | LI | GL | Cecum | 35 | YES | NO |
| DC35 | LI | GL | Cecum | 35 | YES | NO |
| DC11 | LI | FF | Cecum | 35 | YES | YES |
| DC12 | LI | FF | Cecum | 35 | YES | YES |
| DC13 | LI | FF | Cecum | 35 | YES | YES |
| DC14 | LI | FF | Cecum | 35 | YES | YES |
| DC15 | LI | FF | Cecum | 35 | YES | YES |
| DC16 | LI | FF | Cecum | 35 | YES | YES |
| DD41 | SI | XY | Duodenum | 35 | YES | YES |
| DD42 | SI | XY | Duodenum | 35 | YES | YES |
| DD43 | SI | XY | Duodenum | 35 | YES | YES |
| DD44 | SI | XY | Duodenum | 35 | YES | YES |
| DD45 | SI | XY | Duodenum | 35 | YES | YES |
| DD46 | SI | XY | Duodenum | 35 | YES | YES |
| DD21 | SI | RS | Duodenum | 35 | YES | NO |
| DD22 | SI | RS | Duodenum | 35 | YES | NO |
| DD23 | SI | RS | Duodenum | 35 | YES | NO |
| DD24 | SI | RS | Duodenum | 35 | YES | NO |
| DD25 | SI | RS | Duodenum | 35 | YES | NO |
| DD31 | SI | GL | Duodenum | 35 | YES | NO |
| DD32 | SI | GL | Duodenum | 35 | YES | NO |
| DD33 | SI | GL | Duodenum | 35 | YES | NO |
| DD34 | SI | GL | Duodenum | 35 | YES | NO |
| DD35 | SI | GL | Duodenum | 35 | YES | NO |
| DD11 | SI | FF | Duodenum | 35 | YES | YES |
| DD12 | SI | FF | Duodenum | 35 | YES | YES |
| DD13 | SI | FF | Duodenum | 35 | YES | YES |
| DD14 | SI | FF | Duodenum | 35 | YES | YES |
| DD15 | SI | FF | Duodenum | 35 | YES | YES |
| DD16 | SI | FF | Duodenum | 35 | YES | YES |
| DDC42 | LI | XY | Distal colon | 35 | YES | YES |
| DDC43 | LI | XY | Distal colon | 35 | YES | YES |
| DDC44 | LI | XY | Distal colon | 35 | YES | YES |
| DDC45 | LI | XY | Distal colon | 35 | YES | YES |
| DDC46 | LI | XY | Distal colon | 35 | YES | YES |
| DDC21 | LI | RS | Distal colon | 35 | YES | NO |
| DDC22 | LI | RS | Distal colon | 35 | YES | NO |
| DDC23 | LI | RS | Distal colon | 35 | YES | NO |
| DDC24 | LI | RS | Distal colon | 35 | YES | NO |
| DDC25 | LI | RS | Distal colon | 35 | YES | NO |
| DDC31 | LI | GL | Distal colon | 35 | YES | NO |
| DDC32 | LI | GL | Distal colon | 35 | YES | NO |
| DDC33 | LI | GL | Distal colon | 35 | YES | NO |
| DDC34 | LI | GL | Distal colon | 35 | YES | NO |
| DDC35 | LI | GL | Distal colon | 35 | YES | NO |
| DDC12 | LI | FF | Distal colon | 35 | YES | YES |
| DDC13 | LI | FF | Distal colon | 35 | YES | YES |
| DDC14 | LI | FF | Distal colon | 35 | YES | YES |
| DDC15 | LI | FF | Distal colon | 35 | YES | YES |
| DDC16 | LI | FF | Distal colon | 35 | YES | YES |
| DF41 | LI | XY | Feces | 35 | YES | YES |
| DF42 | LI | XY | Feces | 35 | YES | YES |
| DF43 | LI | XY | Feces | 35 | YES | YES |
| DF44 | LI | XY | Feces | 35 | YES | YES |
| DF45 | LI | XY | Feces | 35 | YES | YES |
| DF46 | LI | XY | Feces | 35 | YES | YES |
| DF21 | LI | RS | Feces | 35 | YES | NO |
| DF22 | LI | RS | Feces | 35 | YES | NO |
| DF23 | LI | RS | Feces | 35 | YES | NO |
| DF24 | LI | RS | Feces | 35 | YES | NO |
| DF32 | LI | GL | Feces | 35 | YES | NO |
| DF33 | LI | GL | Feces | 35 | YES | NO |
| DF34 | LI | GL | Feces | 35 | YES | NO |
| DF35 | LI | GL | Feces | 35 | YES | NO |
| DF11 | LI | FF | Feces | 35 | YES | YES |
| DF12 | LI | FF | Feces | 35 | YES | YES |
| DF13 | LI | FF | Feces | 35 | YES | YES |
| DF14 | LI | FF | Feces | 35 | YES | YES |
| DF15 | LI | FF | Feces | 35 | YES | YES |
| DF16 | LI | FF | Feces | 35 | YES | YES |
| DI41 | SI | XY | Ileum | 35 | YES | YES |
| DI42 | SI | XY | Ileum | 35 | YES | YES |
| DI43 | SI | XY | Ileum | 35 | YES | YES |
| DI45 | SI | XY | Ileum | 35 | YES | YES |
| DI46 | SI | XY | Ileum | 35 | YES | YES |
| DI21 | SI | RS | Ileum | 35 | YES | NO |
| DI22 | SI | RS | Ileum | 35 | YES | NO |
| DI23 | SI | RS | Ileum | 35 | YES | NO |
| DI24 | SI | RS | Ileum | 35 | YES | NO |
| DI25 | SI | RS | Ileum | 35 | YES | NO |
| DI31 | SI | GL | Ileum | 35 | YES | NO |
| DI32 | SI | GL | Ileum | 35 | YES | NO |
| DI33 | SI | GL | Ileum | 35 | YES | NO |
| DI34 | SI | GL | Ileum | 35 | YES | NO |
| DI35 | SI | GL | Ileum | 35 | YES | NO |
| DI11 | SI | FF | Ileum | 35 | YES | YES |
| DI12 | SI | FF | Ileum | 35 | YES | YES |
| DI13 | SI | FF | Ileum | 35 | YES | YES |
| DI14 | SI | FF | Ileum | 35 | YES | YES |
| DI15 | SI | FF | Ileum | 35 | YES | YES |
| DI16 | SI | FF | Ileum | 35 | YES | YES |
| DJ41 | SI | XY | Jejunum | 35 | YES | YES |
| DJ42 | SI | XY | Jejunum | 35 | YES | YES |
| DJ43 | SI | XY | Jejunum | 35 | YES | YES |
| DJ44 | SI | XY | Jejunum | 35 | YES | YES |
| DJ45 | SI | XY | Jejunum | 35 | YES | YES |
| DJ46 | SI | XY | Jejunum | 35 | YES | YES |
| DJ21 | SI | RS | Jejunum | 35 | YES | NO |
| DJ22 | SI | RS | Jejunum | 35 | YES | NO |
| DJ23 | SI | RS | Jejunum | 35 | YES | NO |
| DJ24 | SI | RS | Jejunum | 35 | YES | NO |
| DJ25 | SI | RS | Jejunum | 35 | YES | NO |
| DJ31 | SI | GL | Jejunum | 35 | YES | NO |
| DJ32 | SI | GL | Jejunum | 35 | YES | NO |
| DJ33 | SI | GL | Jejunum | 35 | YES | NO |
| DJ34 | SI | GL | Jejunum | 35 | YES | NO |
| DJ35 | SI | GL | Jejunum | 35 | YES | NO |
| DJ11 | SI | FF | Jejunum | 35 | YES | NO |
| DJ12 | SI | FF | Jejunum | 35 | YES | YES |
| DJ13 | SI | FF | Jejunum | 35 | YES | YES |
| DJ14 | SI | FF | Jejunum | 35 | YES | YES |
| DJ15 | SI | FF | Jejunum | 35 | YES | YES |
| DJ16 | SI | FF | Jejunum | 35 | YES | YES |
| DMC41 | LI | XY | Mid colon | 35 | YES | YES |
| DMC42 | LI | XY | Mid colon | 35 | YES | YES |
| DMC43 | LI | XY | Mid colon | 35 | YES | YES |
| DMC45 | LI | XY | Mid colon | 35 | YES | YES |
| DMC46 | LI | XY | Mid colon | 35 | YES | YES |
| DMC21 | LI | RS | Mid colon | 35 | YES | NO |
| DMC22 | LI | RS | Mid colon | 35 | YES | NO |
| DMC23 | LI | RS | Mid colon | 35 | YES | NO |
| DMC24 | LI | RS | Mid colon | 35 | YES | NO |
| DMC25 | LI | RS | Mid colon | 35 | YES | NO |
| DMC31 | LI | GL | Mid colon | 35 | YES | NO |
| DMC32 | LI | GL | Mid colon | 35 | YES | NO |
| DMC33 | LI | GL | Mid colon | 35 | YES | NO |
| DMC34 | LI | GL | Mid colon | 35 | YES | NO |
| DMC35 | LI | GL | Mid colon | 35 | YES | NO |
| DMC11 | LI | FF | Mid colon | 35 | YES | YES |
| DMC12 | LI | FF | Mid colon | 35 | YES | YES |
| DMC13 | LI | FF | Mid colon | 35 | YES | YES |
| DMC14 | LI | FF | Mid colon | 35 | YES | YES |
| DMC15 | LI | FF | Mid colon | 35 | YES | YES |
| DMC16 | LI | FF | Mid colon | 35 | YES | YES |
| DPC41 | LI | XY | Proximal colon | 35 | YES | YES |
| DPC42 | LI | XY | Proximal colon | 35 | YES | YES |
| DPC43 | LI | XY | Proximal colon | 35 | YES | YES |
| DPC44 | LI | XY | Proximal colon | 35 | YES | YES |
| DPC45 | LI | XY | Proximal colon | 35 | YES | YES |
| DPC46 | LI | XY | Proximal colon | 35 | YES | YES |
| DPC21 | LI | RS | Proximal colon | 35 | YES | NO |
| DPC22 | LI | RS | Proximal colon | 35 | YES | NO |
| DPC23 | LI | RS | Proximal colon | 35 | YES | NO |
| DPC24 | LI | RS | Proximal colon | 35 | YES | NO |
| DPC25 | LI | RS | Proximal colon | 35 | YES | NO |
| DPC31 | LI | GL | Proximal colon | 35 | YES | NO |
| DPC32 | LI | GL | Proximal colon | 35 | YES | NO |
| DPC33 | LI | GL | Proximal colon | 35 | YES | NO |
| DPC34 | LI | GL | Proximal colon | 35 | YES | NO |
| DPC35 | LI | GL | Proximal colon | 35 | YES | NO |
| DPC11 | LI | FF | Proximal colon | 35 | YES | YES |
| DPC12 | LI | FF | Proximal colon | 35 | YES | YES |
| DPC13 | LI | FF | Proximal colon | 35 | YES | YES |
| DPC14 | LI | FF | Proximal colon | 35 | YES | YES |
| DPC15 | LI | FF | Proximal colon | 35 | YES | YES |
| DPC16 | LI | FF | Proximal colon | 35 | YES | YES |
