# Supplementary material for: Xylan alleviates dietary fiber deprivation-induced dysbiosis by selectively promoting Bifidobacterium pseudocatenulatum in pigs
Source: Microbiome. 2021 Nov 21;9:227. doi: 10.1186/s40168-021-01175-x (PMC8606072; doi:10.1186/s40168-021-01175-x)
Supplement: Supplementary file 6 — Additional file 5. Figure S1 Experimental protocol. [file 40168_2021_1175_MOESM6_ESM.pdf]

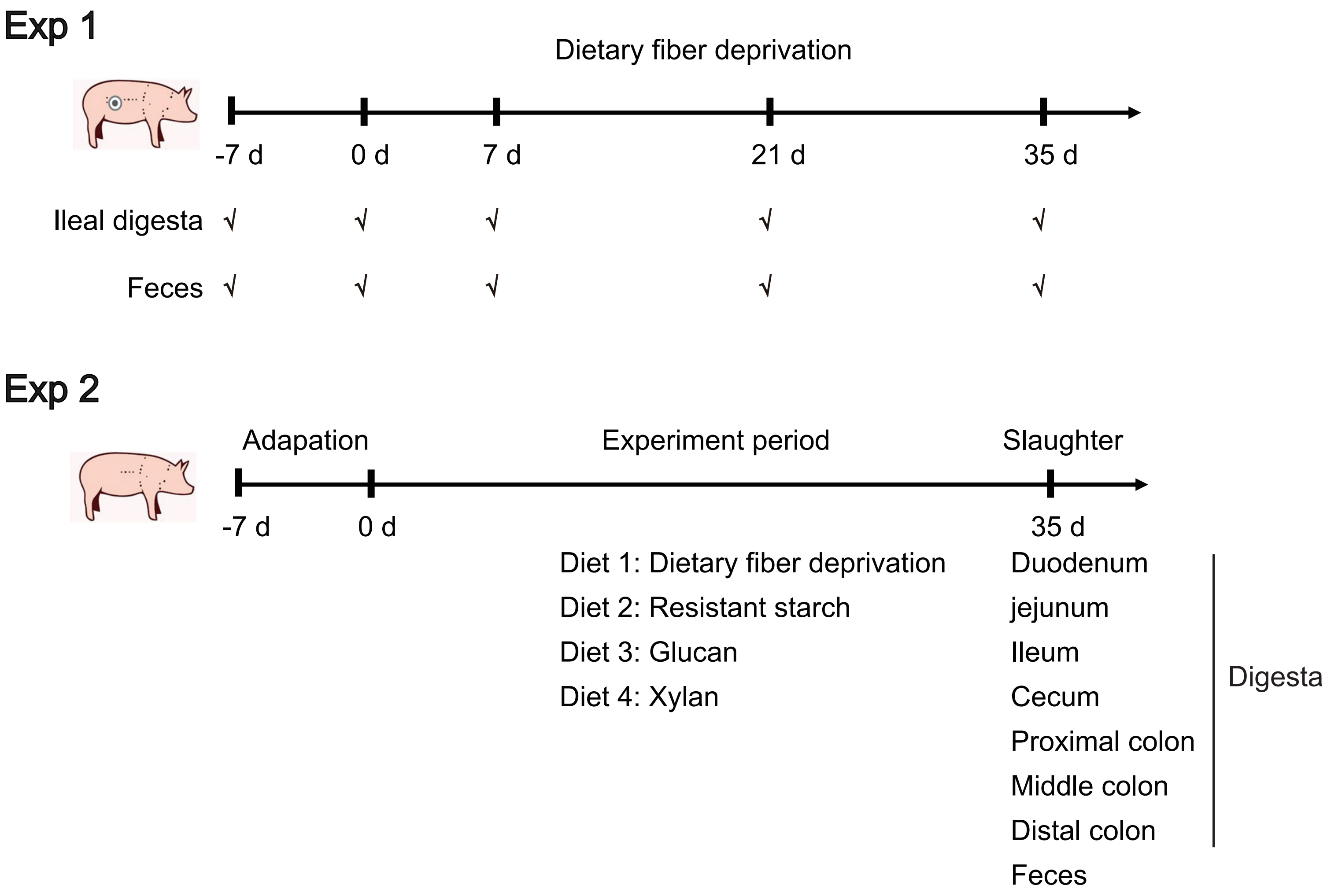
Exp 1
Dietary fiber deprivation
-7 d
0 d
7 d
21 d
35 d
Ileal digesta √ √ √ √ √
Feces √ √ √ √ √
Exp 2
Adapation
Experiment period
Slaughter
-7 d
0 d
35 d
Diet 1: Dietary fiber deprivation
Diet 2: Resistant starch
Diet 3: Glucan
Diet 4: Xylan
Duodenum
jejunum
Ileum
Cecum
Proximal colon
Middle colon
Distal colon
Digesta
Feces
