# Supplementary figures and images for: Xylan alleviates dietary fiber deprivation-induced dysbiosis by selectively promoting Bifidobacterium pseudocatenulatum in pigs
Source: Microbiome. 2021 Nov 21;9:227. doi: 10.1186/s40168-021-01175-x (PMC8606072; doi:10.1186/s40168-021-01175-x)

**A**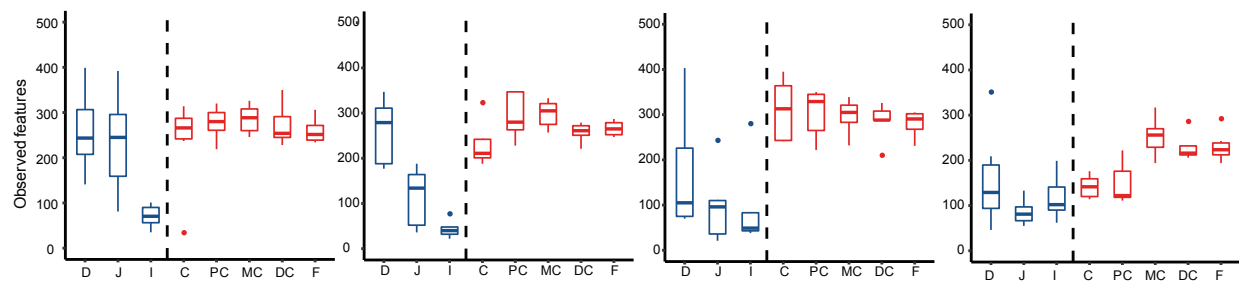**B**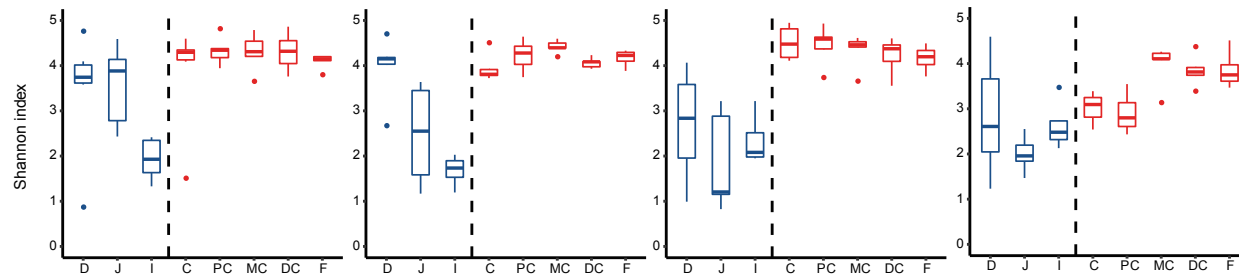

Supplement: Supplementary file 7 — Additional file 6. Figure S2 α-diversity difference between small intestine and large intestine following xylan, β-glucan and resistant starch intervention. D, duodenum; J, jejunum; I, ileum; C, cecum; PC, proximal colon; MC, middle colon; DC, distal colon; F, feces. [file 40168_2021_1175_MOESM7_ESM.pdf]

**A**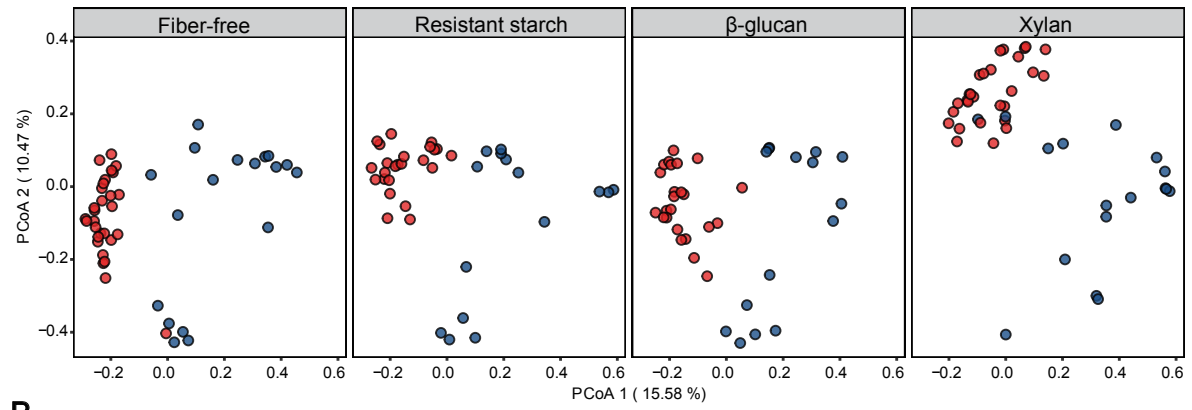**B**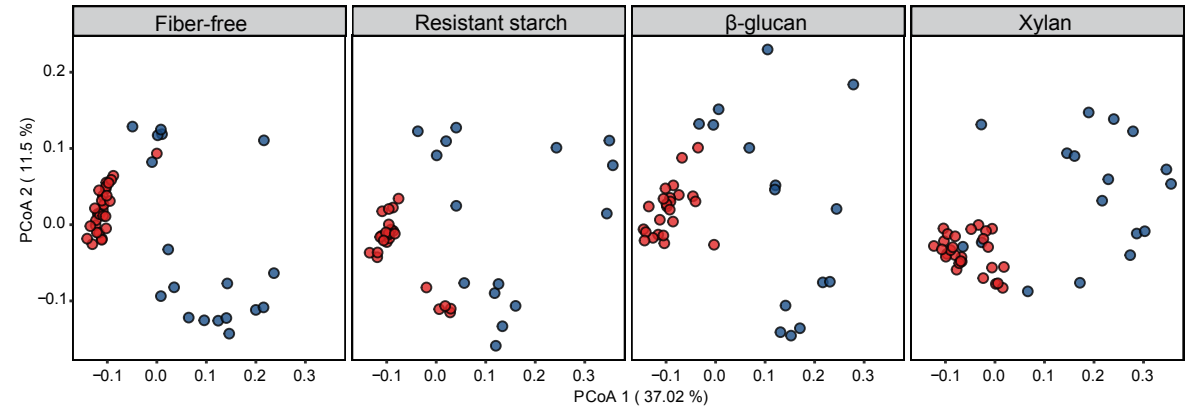

Supplement: Supplementary file 8 — Additional file 7. Figure S3 Community structure difference between small intestine and large intestine following dietary fiber deprivation, xylan, β-glucan and resistant starch intervention based on weighted bray-curtis (A) and unifrac (B) distance metrics. [file 40168_2021_1175_MOESM8_ESM.pdf]

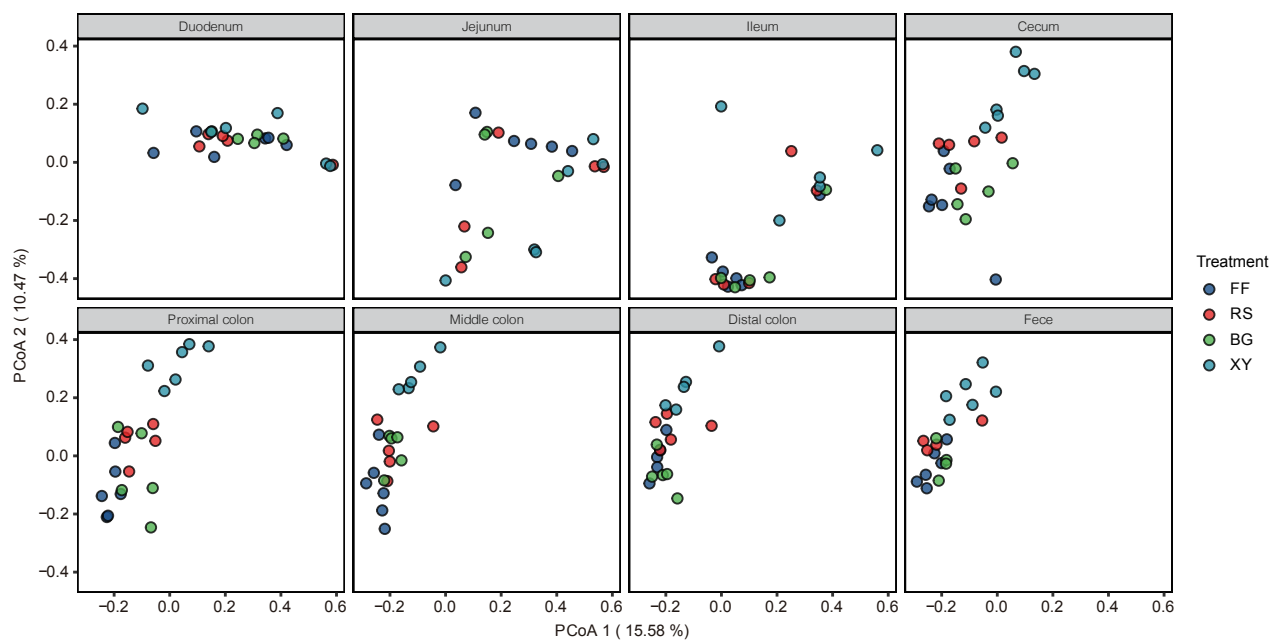

Supplement: Supplementary file 9 — Additional file 8. Figure S4 Community structure along the gastrointestinal tract following dietary fiber deprivation, xylan β-glucan and resistant starch intervention based on weighted bray-curtis distance metrics. FF, dietary fiber deprivation; RS, resistant starch; BG, β-glucan, XY, xylan. [file 40168_2021_1175_MOESM9_ESM.pdf]

**A**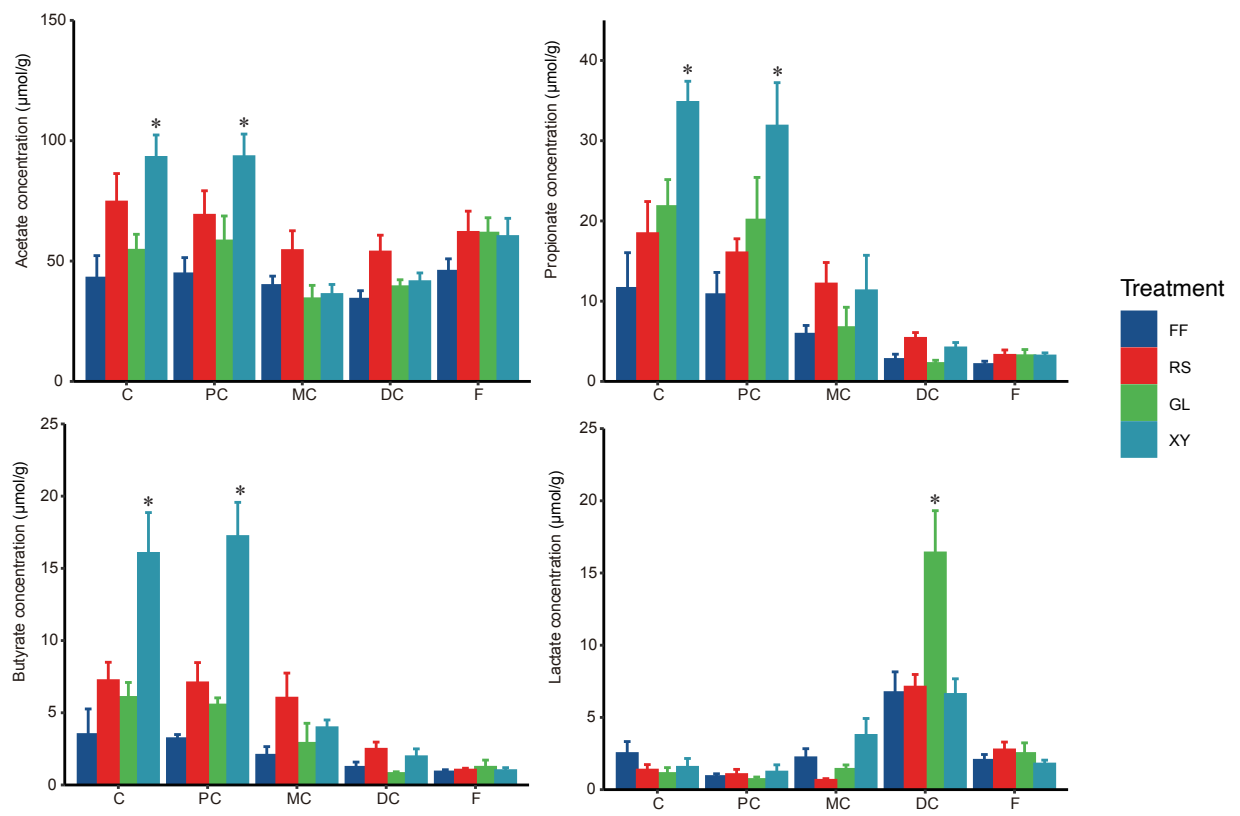

Supplement: Supplementary file 10 — Additional file 9. Figure S5 SCFA concentration within large intestine in response to different treatments. (A) SCFA concentration (μmol/ml wet digesta) within large intestine. Data are represented as means ± SD. *, P < 0.05. FF, dietary fiber deprivation, RS, resistant starch; BG, β-glucan; XY, xylan. [file 40168_2021_1175_MOESM10_ESM.pdf]
